# The biological effect of ^125^I seed continuous low dose rate irradiation in CL187 cells

**DOI:** 10.1186/1756-9966-28-12

**Published:** 2009-01-29

**Authors:** Hong-Qing Zhuang, Jun-Jie Wang, An-Yan Liao, Ji-Dong Wang, Yong Zhao

**Affiliations:** 1 Cancer Center, Department of Radiation Oncology, Peking University Third Hospital, Beijing 100191, PR China; 2 Transplantation Biology Research Division, State Key Laboratory of Biomembrane and Membrane Biotechnology, Institute of Zoology, Chinese Academy of Sciences, Beijing 100101, PR China

## Abstract

**Background:**

To investigate the effectiveness and mechanism of ^125^I seed continuous low-dose-rate irradiation on colonic cell line CL187 in vitro.

**Methods:**

The CL187 cell line was exposed to radiation of ^60^Coγ ray at high dose rate of 2 Gy/min and ^125^I seed at low dose rate of 2.77 cGy/h. Radiation responses to different doses and dose rates were evaluated by colony-forming assay. Under ^125^I seed low dose rate irradiation, a total of 12 culture dishes were randomly divided into 4 groups: Control group, and 2, 5, and 10 Gy irradiation groups. At 48 h after irradiation, apoptosis was detected by Annexin and Propidium iodide (PI) staining. Cell cycle arrests were detected by PI staining. In order to investigate the influence of low dose rate irradiation on the MAPK signal transduction, the expression changes of epidermal growth factor receptor (EGFR) and Raf under continuous low dose rate irradiation (CLDR) and/or EGFR monoclonal antibodies were determined by indirect immunofluorescence.

**Results:**

The relative biological effect (RBE) for ^125^I seeds compared with ^60^Co γ ray was 1.41. Apoptosis rates of CL187 cancer cells were 13.74% ± 1.63%, 32.58% ± 3.61%, and 46.27% ± 3.82% after 2 Gy, 5 Gy, and 10 Gy irradiation, respectively; however, the control group apoptosis rate was 1.67% ± 0.19%. G_2_/M cell cycle arrests of CL187 cancer cells were 42.59% ± 3.21%, 59.84% ± 4.96%, and 34.61% ± 2.79% after 2 Gy, 5 Gy, and 10 Gy irradiation, respectively; however, the control group apoptosis rate was 26.44% ± 2.53%. *P *< 0.05 vs. control groups by Student's t-test were found in every treated group both in apoptosis and in G_2_/M cell cycle arrest. After low dose rate irradiation, EGFR and Raf expression increased, but when EGFR was blocked by a monoclonal antibody, EGFR and Raf expression did not change.

**Conclusion:**

^125^I seeds resulted in more effective inhibition than ^60^Co γ ray high dose rate irradiation in CL187 cells. Apoptosis following G_2_/M cell cycle arrest was the main mechanism of cell-killing effects under low dose rate irradiation. CLDR could influence the proliferation of cells via MAPK signal transduction.

## Background

Because of its ability to offer high precision, little trauma, strong lethality, and fewer complications [1-4], ^125^I radioactive seed implantation has been widely applied in clinical practice for tumor treatment, such as prostate carcinoma [5], recurrent colorectal cancer [6-10], head and neck carcinoma [11,12], and others [13-15]. However, radiobiological study of continuous low dose rate irradiation (CLDR), and especially that which defines the deep development of radioactive seed implantation and its intersection with other subjects of tumor treatment, has only recently been conducted [16,17]. Therefore, further study on the basic radiobiology of continuous low dose rate irradiation is necessary, particularly to provide further clinical direction. In the present study, the CL187 colonic cell line was exposed to ^125^I seeds at low dose rate irradiation, and killing effect of cells cultured in vitro were observed to reveal the radiobilogical mechanism of ^125^I radioactive seed irradiation.

## Materials and methods

### Reagents

Cell culture media was provided by the Zoology Institute of the Chinese Academy of Sciences. Propidium iodide (PI) and annexin V were purchased from Cell Signaling Company (Cell Signaling Technology, Beverly, MA). Phospho-P38 epidermal growth factor receptor (EGFR) mAb (Alexa Fluor) and Phospho-raf mAb (Alexa Fluor) were obtained from Santa Cruz Biotechnology, Inc. (Santa Cruz, CA). All other materials were obtained from the Zoology Institute of the Chinese Academy of Sciences.

### Cell lines and cell culture

The CL187 colonic cancer cell line was kindly provided by the Beijing Institute for Cancer Research. It was maintained in RPMI1640 supplemented with 20 mM HEPES (pH 7.4), 100 IU/mL penicillin, 100 mg/mL streptomycin, 4 mM glutamine, and 10% heat-inactivated fetal bovine serum (Hangzhou Sijiqing Biological Engineering Materials Company, China) in a humidified atmosphere of 95% air and 5% CO2 at 37°C.

### ^125^I seeds irradiation

**Figure 1:**
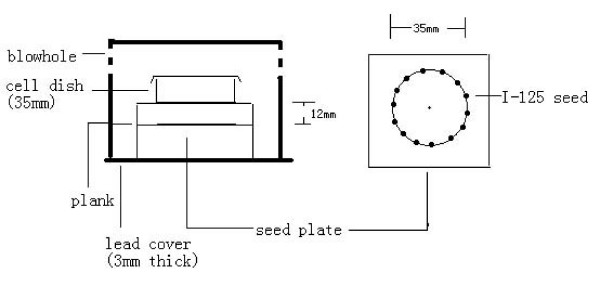
**^125^I seed experiment irradiation pattern in vitro**.

We used our in-house developed in vitro iodine-125 seed irradiation model shown in Figure 1[18]. The model consists of a 3-mm thick polystyrene panel, with a lower seed plaque layer and an upper cell culture plaque layer. In the seed plaque, 14 seeds with the same activity were equally spaced within recesses (4.5 mm × 0.8 mm) around a 35-mm diameter (D) circumference. In the cell culture plaque, the same recesses were made around a 35-mm D circumference; its center was along the same vertical line as that of the seed plaque, so that a 35-mm Petri dish could be placed on it during the experiment. The height (H) between the seed plaque and the bottom of Petri dish was 12 mm, with a D/H ratio of 2.9. The purpose of this design was to obtain a relatively homogeneous dose distribution at the bottom of the Petri dish. The polystyrene assembly was enclosed by a 3-mm thick lead chamber with a vent-hole, so that during the study the whole model could be kept in the incubator. The incubator played a protective role by maintaining constant cell culture conditions. Model 6711 ^125^I seeds were provided by Ningbo Junan Pharmaceutical Technology Company, China. The single seed activity used in this study was 92.5 MBq (2.5 mCi), corresponding initial dose rate in model cells was 2.77 cGy/h. The dose uniformity of the irradiation model in the cell plane was 1.34, which was similar to other investigators' results [2]. The model was validated using thermoluminescent dosimetry (TLD) measurement. The absorbed dose for different exposure time in various culture planes has also been measured and verified. The exposure time for delivering doses of 100, 200, 400, 600, 800 and 1000 cGy are 36, 73.7, 154.6, 245.8, 345.1, 460.1 hours. Exponentially-growing CL187 cells in a tissue-culture flask (35 mm diameter) were irradiated using the above model. The cells were subsequently incubated for another 21 d at constant temperature and humidity. Irradiation was performed at the Zoology Institute of the Chinese Academy of Sciences.

### Clonogenic survival

Clonogenic survival was defined as the ability of cells to maintain clonogenic capacity and to form colonies. Briefly, cells in the control and irradiation groups were exposed to different radiation dosages (0, 1, 2, 4, 6, 8, and 10 Gy). After incubation for 21 d, colonies were stained with crystal violet and manually counted. The plating efficiency (PE) and survival fraction (SF) were calculated as follows: PE = (colony number/inoculating cell number) × 100%. SF = PE (tested group)/PE (0-Gy group) × 100%. A dose-survival curve was obtained for each experiment and used for calculating several survival parameters. Parallel samples were set at each irradiation dosage. The cell-survival curve was plotted with Origin 7.5 software, using the equation: SF = 1 - (1 - e^-D/D0^)^N^. The multi-target, single-hit model was applied to calculate cellular radiosensitivity (mean lethal dose, D_0_), capacity for sublethal damage repair (quasithreshold dose, Dq), and extrapolation number (N). The D_10_values were used to calculate the relative biological effect (RBE).

### Cell cycle and apoptosis analysis

Cells from the control and CLDR-treated groups were exposed to different radiation dosages (0, 2, 5, and 10 Gy). Cells were harvested 48 h after irradiation. For detection of apoptotic cells, cells were trypsinized, acridine orange stained, and determined under fluorescence microscope. At the same time, cells were counted and washed twice with cold PBS. Cells used for apoptosis tests were stained with propidium iodide (PI) and annexin V for 15 min in the dark. Cells used for cell-cycle testing were stained with propidium iodide after ethanol fixation and analyzed by fluorescence-activated cell sorting (FACS) using Coulter EPICS and ModFit software (Verity Software House, Topsham, MN). Each test was performed 3 times [19].

### EGFR and Raf quantifications by FCM

Control and treated CL187 cells for EGFR and Raf quantifications by FCM were harvested 24 h after 4 Gy irradiation. Each test was performed 3 times. Cells used for tests were stained with Phospho-P38 EGFR mAb (Alexa Fluor) and Phospho-raf mAb (Alexa Fluor), and then analyzed by FACScan using Coulter EPICS and ModFit software. Each test was performed 3 times [20-22].

### Statistical analysis

Data were plotted as means ± standard deviation. Student's t test was used for comparisons. Differences were considered significant at P < 0.05.

## Results

### Survival curve of CL187 cells after different dose rate irradiation

**Figure 2:**
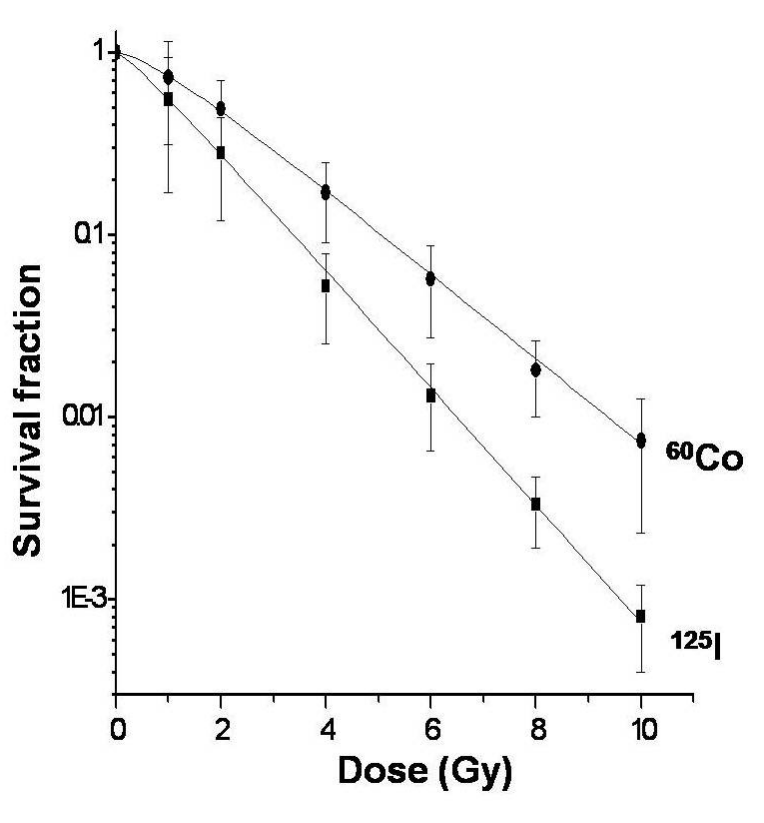
**Dose-survival curves of CL187 cells after high and low dose rate irradiation**.

Data showed that cell-killing effects were related to dose rate. The survival curve of CL187 cells after different dose rate irradiation is shown in Figure 2. At the same dose, the survival fractions of ^125^I seeds were always lower than ^60^Co γ ray (Table 1). The cloning efficiency of CL187 was between 70% and 90%. Radiobiological parameters of high dose rate irradiation treated CL187 cells were D_0 _= 1.85, Dq = 0.35, and N = 1.55, while those of ^125^I seed low dose rate irradiation cells were D_0 _= 1.32, Dq = 0.14, and N = 1.28. In the present study, RBE = D_10 _^60^Co/D_10_^125^I = 4.23/3.01 = 1.41. The data presented herein suggested that the biological effect of ^125^I seed irradiation was stronger than that of ^60^Co γ ray (t = 2.578, *P *< 0.05).

**Table 1:** Survival fraction of different dose rate irradiation in CL187 cell line (%, $\bar{x}$ ± s)

	Irradiation dose (Gy)					
	
	1	2	4	6	8	10
Survival fraction						
^60^Co	73 ± 22	49 ± 11	17 ± 5.2	5.7 ± 2.1	1.8 ± 0.19	0.74 ± 0.21
^125^I	55 ± 18^a^	28 ± 10^b^	5.2 ± 2.7^c^	1.3 ± 0.25^d^	0.33 ± 0.12^e^	0.08 ± 0.03^f^

Compared with ^60^Co group, t = 8.03, ^a^P < 0.05; t = 4.85, ^b^P < 0.05; t = 13.69, ^c^P < 0.01; t = 11.43, ^d^P < 0.01; t = 4.76, ^e^P < 0.05; and t = 4.62, ^f^P < 0.05.

### Low dose rate irradiation-induced apoptosis and cell cycle arrest

**Figure 3:**
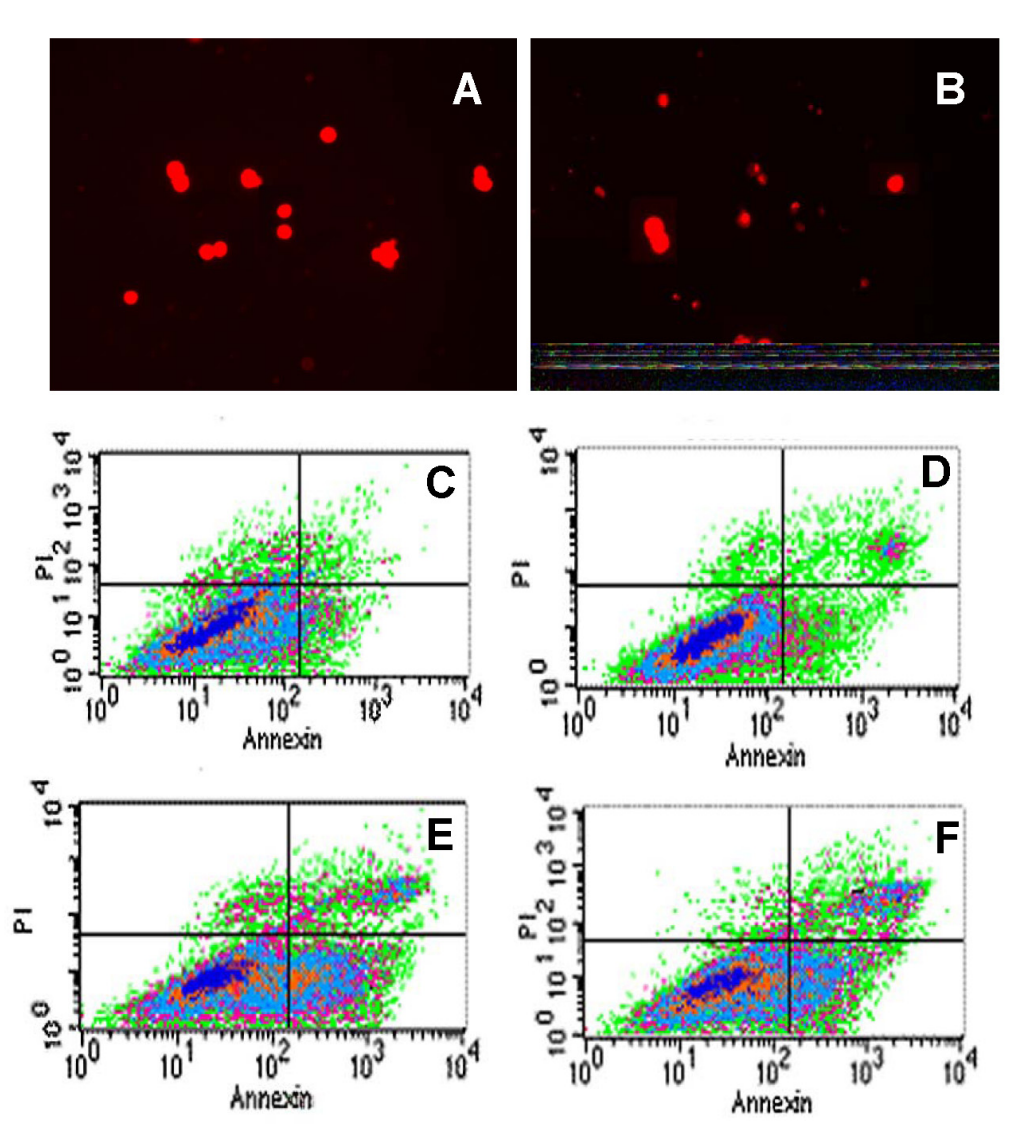
**Apoptosis of ^125^I low dose rate irradiation-treated CL187 cells**. CL187 cells were stained with acridine orange, and determined under fluorescence microscope. There were no apoptotic cells in control groups (A), but typical morphological features of apoptosis appeared after 5 Gy CLDR irradiation (B). The apoptotic rates were detected by flow cytometry. In 2 Gy (D), 5 Gy (E), and 10 Gy (F) groups, the CL187 cells had higher apoptosis rates when compared with control groups (C). Concrete data see table 3. One of three experiments is shown. *P *< 0.05 vs. control group were found in every treated groups.

**Figure 4:**
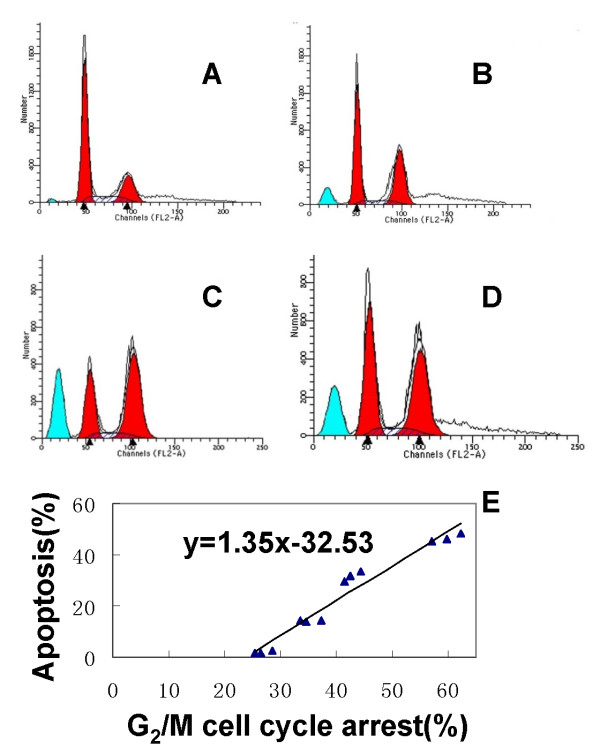
**Effect of ^125^I low dose rate irradiation on the cell cycle in CL187 cells**. Flow cytometry analysis revealed that the G_2_/M phase increased by 2 Gy (B)^125I ^irradiation dose as compared with untreated control cells (A). After 5 Gy irradiation (C), a sharp increase in the fraction of cells in the G_2_/M phase was observed. The result in 10 Gy irradiation groups (D) was lower than that in group C, but sustained at a relatively high level. Compared with untreated control cells, *P *< 0.05 were found in all of the treated groups.

Cells were stained by acridine orange and observed under fluorescence microscopy; typical morphological features of apoptotic cells appeared after 5 Gy low dose rate irradiation (Fig. 3). FCM analysis showed that under low dose rate irradiation, apoptosis and G_2_/M cell cycle arrest increased slightly at 2 Gy, the peak appeared at 5 Gy, and the ratio was also high at 10 Gy (Table 2) but lower than that at 5 Gy. Furthermore, G_2_/M cell cycle arrest and apoptosis walked together along with the dose change (r = 0.918, *P *< 0.01, Fig. 4). Quantitative measurements of apoptotic cell death by FCM in CL187 cells sufficiently indicated that apoptosis is an important mechanism of low dose rate irradiation inhibition of CL187 cell proliferation.

**Table 2:** Apoptosis index and cell cycle distribution after ^125^I low dose rate irradiation (%, $\bar{x}$ ± s).

	Apoptosis	G_0_/G_1_	S	G_2_/M
Control	1.67 ± 0.19	64.94 ± 5.87	8.62 ± 0.59	26.44 ± 2.53
2 Gy	13.74 ± 1.63^a^	54.14 ± 3.16	11.25 ± 1.34	34.61 ± 2.79^d^
5 Gy	46.27 ± 3.82^b^	26.60 ± 2.82	13.56 ± 1.68	59.84 ± 4.96^e^
10 Gy	32.58 ± 3.61^c^	41.69 ± 4.58	15.72 ± 2.29	42.59 ± 3.21^f^

Compared with control group (apoptosis), t = 8.377, ^a^P < 0.05; t = 36.44, ^b^P < 0.01; and t = 27.35, ^c^P < 0.01. Compared with control group (G_2_/M arrests), t = 30.81, ^d^P < 0.05; t = 23.98, ^d^P < 0.05; and t = 26.3, ^e^P < 0.05.

### Expression changes of EGFR and Raf in CL187 cells after irradiation and/or EGFR monoclonal antibody treatment

**Figure 5:**
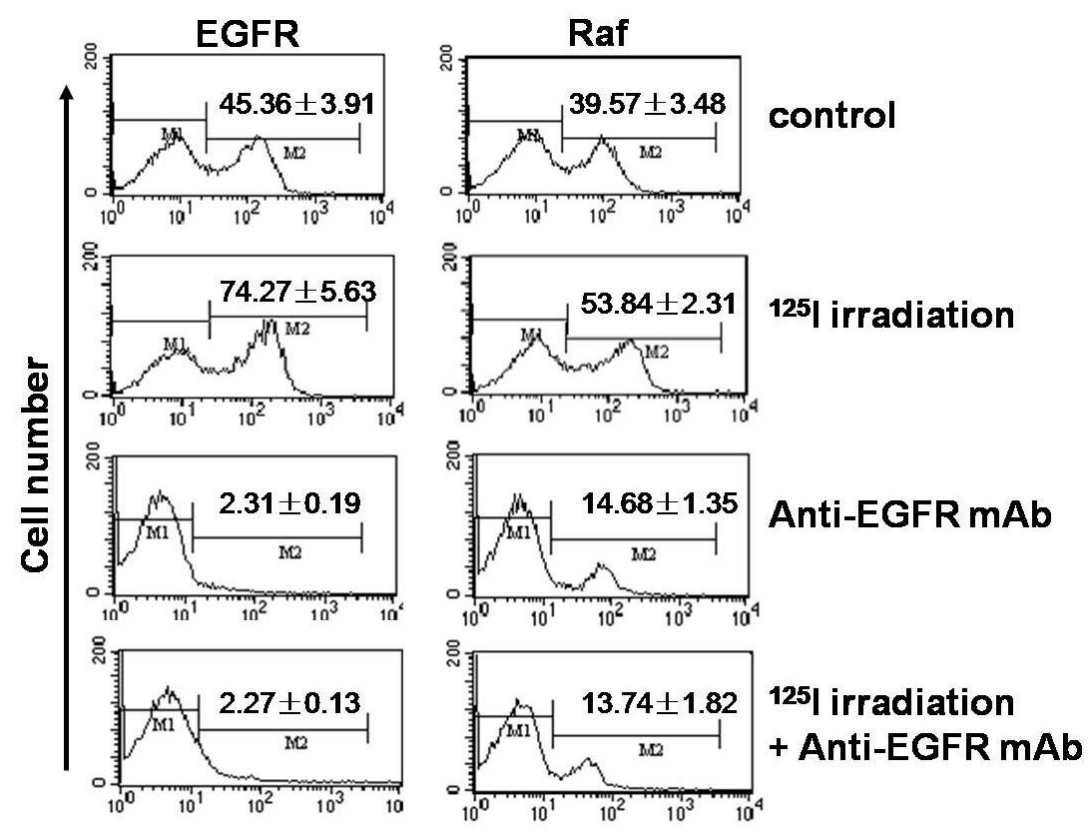
**EGFR and Raf expression changes in CL187 cells after ^125^I irradiation and/or Anti-EGFR mAb**. CLDR could influence the proliferation of cells via MAPK signal transduction. One representive of two experiments is shown.

Under low dose rate irradiation, expression of EGFR (74.27 ± 5.63%) and Raf (53.84 ± 2.31%) was significantly higher than in the control group (Fig. 5 and Table 3). After signal transduction was blocked, expression of EGFR (2.07 ± 0.31%) and Raf (13.74 ± 1.82%) did not show detectable change after low dose rate irradiation (Fig. 5 and Table 3).

**Table 3:** Expression changes of EGFR and Raf in CL187 cells after irradiation and/or EGFR monoclonal antibody treatment (%, $\bar{x}$ ± s).

	EGFR	Raf
Control	45.36 ± 3.91	39.57 ± 3.48

^125^I irradiation	74.27 ± 5.63^a^	53.84 ± 2.31^d^
Anti-EGFR mAb	2.31 ± 0.19^b^	14.68 ± 1.35^e^
^125^I irradiation + Anti-EGFR mAb	2.27 ± 0.13^c^	13.74 ± 1.82^f^

Compared with control group (EGFR), t = 54.84, ^a^P < 0.01; t = 27.38, ^b^P < 0.05. Compared with anti-EGFR mAb group (EGFR), t = 1.21, ^c^P > 0.05. Compared with control group (Raf), t = 46.66, ^d^P < 0.01; and t = 26.60, ^e^P < 0.01. Compared with anti-EGFR mAb group (Raf), t = 0.98, ^f^P > 0.05.

## Discussion

Low-energy radioactive seed interstitial implantation has resulted in positive clinical treatment of many tumors previously radioresistant to high dose rate irradiation. This may be due to different radiobiological mechanisms between low and high dose rate irradiation. Nevertheless, compared with springing up of radioactive seeds interstitial implantation, fundamental research on this topic is notably absent, and the radiobiological mechanism of ^125^I seed low dose rate irradiation remains unclear.

As classic methods of appraising killing efficacy of irradiation, cell proliferation and clonic assays were used in the experiment. High dose rate irradiation killed tumor cells, but simultaneously induced radioresistance. However, the dose survival curve of ^125^I seed continuous low dose rate irradiation had no significant shoulder region, and SF was lower than ^60^Co γ ray high dose rate irradiation. From the radiobiological parameter results, we also observed that ^125^I continuous low dose rate irradiation showed great advantages relative to high dose rate irradiation. Although RBE could be affected by many factors, such as cell line and dose rate, most studies have shown that the RBE of ^125^I was between 1.3 and 1.5. The present results are consistent with previous reports [24-27].

Our results indicated that apoptosis may play a central role regarding the observed killing effects when cells were exposed to ^125^I seed low dose rate irradiation [28,29]. Prior studies have suggested that radiosensitivity is cell cycle dependent, and cells in the G_2_/M phase could be more radioresponsive [30]. These results suggest that CLDR may enhance radiosensitivity by inducing accumulation of cells in a more radiosensitive cell cycle phase (G_2_/M) [31,32]. The apoptosis index of 10 Gy was lower than that of 5 Gy; two possibilities for this occurrence are: (a) Early-apoptotic cells disintegrated within the exposure time of 10 Gy, and could not be detected by FCM; and (b) Low dose rate irradiation only delayed the cell cycle, but could not completely block the cell cycle. Overshoot early irradiation, cells changed to be more radioresistant. Therefore, the apoptotic cells under 10 Gy were fewer than those under 5 Gy. Similarly, G_2_/M arrest also declined under 10 Gy [33].

Our results indicated that the up-regulation of Raf expression correlated well with an increase in the level of EGFR expression after ^125^I seed irradiation [34-37]. It is suggested that the expression changes were all induced by CLDR. It is essential to prove that CLDR functioned via MAPK signal transduction. When the signal transduction was blocked by the EGFR monoclonal antibody, no obvious change in Raf expression occurred after ^125^I seed irradiation. It was proved that the necessary conditions were also sufficient [38,39]. These results formed the basis for combining CLDR with EGFR tyrosine kinase inhibitors in clinical practice [40,41,22].

In summary, our study provides a beneficial exploration of radiobiology of continuous low dose rate irradiation. Although many issues remain to be addressed, we believe that, with further development of fundamental research, application of ^125^I radioactive seed implantation in clinical practice will continue to be improved.

## Abbreviations

LDR: low-dose rate; HDR: high-dose rate; SLD: sublethal damage; SSB: single strand breaks; DSB: double strand breaks; ATM: ataxia-telangiectasia mutated; NHEJ: nonhomologous end joining; HR: homologous recombination; HRS: hyper-radiosensitivity; RBE: relative biological effectiveness; PE: plating efficiency; SF: survival fraction; CLDR: continuous low-dose-rate; EGFR: epidermal growth factor receptor.

## Competing interests

The authors declare that they have no competing interests.

## Authors' contributions

HQZ carried out cell colony-forming assay, fluorescence-activated cell sorting, flow cytometric analysis, and drafted the manuscript. JJW participated in its design and revised the manuscript. AYL performed the statistical analysis. JDW carried out the irradiation experiment. YZ supervised experimental work and revised the manuscript. All authors read and approved the final manuscript.
